# Food Additive Titanium Dioxide (E171) Increases Intracellular Labile Fe^2+^ Levels and Induces Oxidative Stress and Mitochondrial Dysfunction in H9c2 Cardiomyoblasts

**DOI:** 10.3390/jox16030108

**Published:** 2026-06-09

**Authors:** Alfredo Cruz-Gregorio, Alejandro Silva-Palacios, Javier A. Belmont-Díaz, María del Pilar Ramos-Godinez, Rebeca López-Marure

**Affiliations:** 1 Departamento de Fisiología, Instituto Nacional de Cardiología Ignacio Chávez, Mexico City 14080, Mexico; rebeca.lopez@cardiologia.com.mx; 2 Departmento de Biomedicina Cardiovascular, Instituto Nacional de Cardiología Ignacio Chávez, Mexico City 14080, Mexico; alejandro.silva@cardiologia.org.mx; 3 Departamento de Bioquímica, Instituto Nacional de Cardiología Ignacio Chávez, Mexico City 14080, Mexico; javier.belmont@cardiologia.org.mx; 4 Departamento de Microscopía Electrónica, Instituto Nacional de Cancerología, Mexico City 14080, Mexico; mramosg@incan.edu.mx; 5 Centro de Investigación, Facultad de Medicina UAM-UABJO, Facultad de Medicina y Cirugía, Universidad Autónoma “Benito Juárez” de Oaxaca, Oaxaca 68020, Mexico

**Keywords:** E171, cardiomyoblasts, iron metabolism, oxidative stress, mitochondrial dysfunction, quercetin

## Abstract

Food-grade titanium dioxide (E171) is widely used as a food additive and has raised concerns about its potential systemic toxicity. However, its impact on cardiac cellular metabolism and mitochondrial function remains incompletely understood. In the present study, we investigated the effects of E171 on intracellular labile iron, oxidative stress, mitochondrial ultrastructure, and mitochondrial bioenergetics in H9c2 cardiomyoblasts. Exposure to E171 leads to a significant increase in intracellular labile iron (Fe^2+^) levels. This alteration was accompanied by elevated reactive oxygen species (ROS) production and reduced intracellular glutathione levels, consistent with enhanced oxidative stress following E171 exposure. Ultrastructural analysis by transmission electron microscopy (TEM) revealed marked mitochondrial alterations, including reduced cristae density and structural damage. Functional assessment of mitochondrial bioenergetics demonstrated impaired oxidative phosphorylation and reduced maximal respiratory capacity in E171-treated cells. The potential protective role of quercetin (a powerful antioxidant and iron chelator) was explored in mitochondrial respiration assays; however, at the concentration and exposure conditions tested, quercetin treatment did not fully restore the bioenergetic parameters induced by E171. Collectively, these findings indicate that E171 increases intracellular labile iron levels and promotes oxidative stress, associated with alterations in mitochondrial ultrastructure and impaired bioenergetic function in H9c2 cardiomyoblasts, suggesting a potential mechanism by which food additives may affect cardiac cellular metabolism.

## 1. Introduction

Food additives are used in the production of ultra-processed products in the modern human diet. There is evidence that emulsifiers, sweeteners, colorings, and micro- and nanoparticles could have adverse effects, raising growing concerns about their potential negative effects on the health of consumers of these products [1]. Among these, food additive E171 is widely used as a whitening agent in the food industry and is commonly present in processed products. E171 is composed of around 40% nanoparticles and 60% microparticles [2]. Due to its nanometric size, E171, upon ingestion, enters the intestine and, within the intestinal villi, is translocated into the bloodstream, reaching all systems and organs [3]. Although titanium dioxide nanoparticles have long been considered relatively inert, recent studies suggest that exposure to them may induce oxidative stress and cellular dysfunction across diverse biological systems [4]. However, the mechanisms underlying these effects remain incompletely understood, particularly in cells with high metabolic demand such as cardiomyocytes [5].

A growing body of evidence indicates that E171 exposure can disrupt cellular redox homeostasis and promote the generation of reactive oxygen species (ROS) [6], which, when present in excess, can overwhelm endogenous antioxidant systems, leading to oxidative damage to proteins, lipids, and organelles [7]. Mitochondria are especially vulnerable to oxidative stress because they represent both a major source and target of ROS [8]. In addition, alterations in intracellular iron levels may further exacerbate oxidative damage, as iron can catalyze the formation of highly reactive radicals that impair mitochondrial structure and function [9].

Cardiomyocytes rely heavily on mitochondrial bioenergetics to sustain contractile activity, making them particularly sensitive to disturbances in redox balance and mitochondrial integrity [10]. The latter may result from increased iron accumulation, which induces the generation of ROS, thereby promoting an imbalance between oxidative and antioxidant systems. Since mitochondria are dependent on redox homeostasis, a rupture can induce mitochondrial dysfunction [9]. Despite increasing interest in the biological effects of engineered nanoparticles, relatively little is known about the influence of E171 on intracellular iron levels and mitochondrial function in cardiomyocytes. Therefore, the present study aimed to investigate whether exposure to E171 disrupts intracellular iron balance, promotes oxidative stress, and impairs mitochondrial ultrastructure and mitochondrial bioenergetics in H9c2 rat cardiomyoblasts. Quercetin (Q) has been widely reported to modulate iron homeostasis and oxidative stress in various cellular models [11]. Based on these properties, we evaluated whether Q could preserve mitochondrial functional parameters, particularly mitochondrial respiration, under nanoparticle-induced stress conditions. However, Q treatment did not significantly ameliorate the bioenergetic alterations induced by E171 exposure at the concentration and exposure conditions evaluated, suggesting that mitochondrial dysfunction in this context may occur through mechanisms that are not fully reversible by redox modulation alone.

## 2. Materials and Methods

### 2.1. Chemicals

Potassium chloride (KCl), ethanesulfonic acid (HEPES), calcium chloride (CaCl_2_), magnesium chloride (MgCl_2_), glucose, 5,5-dithio-bis (2 nitrobenzoic) acid (DTNB), glutathione reduced (GSH), glutathione oxidized (GSSG), glutathione reductase (GR), dihydroethidium (DHE), 2,7-dichlorofluorescin diacetate (H_2_DCFDA), potassium phosphate (K_2_HPO_4_), 5,5′-dithiobis(2-nitrobenzoic acid) (DTNB), carbonyl cyanide chlorophenyl hydrazone (CCCP), β-nicotinamide adenine phosphate dinucleotide (NADPH), sodium succinate dibasic, dimethyl sulfoxide (DMSO), sodium phosphate dibasic (Na_2_HPO_4_), sodium phosphate monobasic (NaH_2_PO_4_), triethanolamine, 2-vinylpyridine (2-VP), ferrous sulfate (FeSO_4_), quercetin (Q) and Dulbecco’s Modified Eagle’s Medium (DMEM) medium were purchased from Sigma-Aldrich (St. Louis, MO, USA). Fetal bovine serum (FBS) was obtained from Hi-Media (Thane (West), Maharashtra, India).

### 2.2. Cell Culture of H9c2

The H9c2 epithelial cell line (CRL-1446™) was purchased from American Type Culture Collection (ATCC). H9c2 was maintained in DMEM supplemented with 10% FBS at 37 °C and 5% carbon dioxide (CO_2_) in a humidified incubator. Passages 9–15 were used in experiments. H9c2 cardiomyoblasts were exposed to 20 µg/cm^2^ of E171 dispersed in complete culture medium for 24 h. Control cells were maintained under identical conditions without exposure to E171. Non-exposed cells were used as a negative control. We used H9c2 cells because they are an excellent model of cardiac muscle cells, exhibiting many of their properties. Thus, we first aimed to determine the effect of E171 in vitro to inform future studies in rodent models.

### 2.3. Overall Levels of Labile Iron (II) Ions (Fe^2+^)

Fe^2+^ produces harmful ROS upon contact with oxygen, including superoxide (O_2_•^−^), and hydrogen peroxide (H_2_O_2_), which are major contributors to oxidative damage in cells. BioTracker™ FerroOrange Live Cell Dye is a fluorescent probe that specifically detects Fe^2+^ only. Interestingly, the fluorescence intensity does not increase in the presence of iron (III) ions (Fe^3+^) or divalent metal ions other than iron. It also does not react to chelated iron in ferritin and other substances. Thus, we used 1 μM of this dye to measure Fe^2+^ levels. Fe^2+^ was identified by orange fluorescent signals under the microscope and then quantified by fluorometry. In brief, we treated 174,545 H9c2 cells with E171 overnight, the culture medium was removed, and the cells were gently washed twice with PBS to remove extracellular Fe^2+^. Then, 1 μM of dye was added to the cells, and the cells were incubated at 37 °C for 30 min. After staining, the cells were rinsed twice with phosphate-buffered saline (PBS). Thus, Fe^2+^ was visualized and measured with excitation at 510 nm and emission at 590 nm. Quantitative data on ROS and cell images were collected using the LionHeart FX™ from Agilent BioTek (Winooski, VT, USA) at 20×, which combines digital wide-field microscopy with a conventional multi-mode microplate, providing high sensitivity for Fe^2+^ quantification. FeSO_4_ was used as a positive control to generate intracellular Fe^2+^ and to validate the assay’s sensitivity.

### 2.4. ROS Production

ROS production was measured using two fluorescent probes: DHE (red signal) and H_2_DCFDA (green signal). DHE is oxidized to 2-hydroxyethidium and ethidium (Et) in the cytosol by O_2_•^−^. Both compounds are then retained in the nucleus, which is then stained due to their DNA-binding capacity. H_2_DCFDA is a cell-permeable non-fluorescent probe that is de-esterified intracellularly and turns into highly fluorescent dichlorofluorescein (DCF) upon oxidation by ROS. Subsequently, ROS were identified as red- and green-fluorescent signals under the microscope and quantified by fluorometry. In a nutshell, 174,545 H9c2 cells treated with E171 were seeded, and after 24 h, 15 μM DHE and 15 μM H_2_DCFDA were independently added to the culture medium without phenol red and incubated for 30 min at 37 °C. ROS production was visualized and measured at 510 nm excitation and 590 nm emission for DHE and at 480 nm excitation and 520 nm emission for DCF, using the LionHeart FX™ from Agilent BioTek at a magnification of 20×. The quantitative data of ROS and cell images were collected in three independent experiments, which were examined using Gen5 3.0 software.

### 2.5. Glutathione Quantification (GSH/GSSG Ratio)

Total glutathione (GSH + GSSG) and oxidized glutathione (GSSG) were determined using the enzymatic recycling method as described by Rahman et al. (2006) [12], in which GSH is oxidized by DTNB to 5-thio-2-nitrobenzoic acid (TNB, detectable at λ = 412 nm) and glutathione-TNB adducts (GSH-TNB). Both GSH-TNB and GSSG are reduced by GR in the presence of NADPH to GSH, which in turn is oxidized by DTNB to TNB. In this manner, the total glutathione amount calculated in this first step represents the sum of GSH and GSSG. Next, GSSG was selectively quantified using the enzymatic recycling method described above, in which samples were previously treated with 2-VP. 2-VP, which can covalently associate with GSH, removes GSH, leaving GSSG as the only measurable substrate of the assay. Briefly, 2-VP was diluted 1:10 (*v*/*v*) immediately prior to use, and 2 µL of this dilution was added to 100 µL of sample extract. Samples were incubated at room temperature for 1 h to ensure effective masking of reduced GSH prior to enzymatic measurement. Finally, GSH was calculated as the difference between GSSG and total glutathione (GSH + GSSG). Glutathione levels were normalized to total protein content, quantified by the Bradford assay, and expressed as nmol/mg protein.

Briefly, the H9c2 cell extracts were diluted with 120 μL of 0.1 M K_2_HPO_4_, 5 mM disodium EDTA buffer, pH 7.5. Then, two separate 20 μL samples were used to measure either GSH + GSSG or GSSG (these samples had been previously treated with 2-VP) and were mixed with 2.5 mM DTNB and 250 U/mL GR. Finally, β-NADPH was added, and absorbance at λ = 412 nm was measured every 60 s for 2 min. The rate of change in absorbance for each experiment was compared with GSH or GSSG standards.

### 2.6. H9c2 Cell Respirometry

Oxygen consumption rates (OCR) in intact H9c2 cells exposed to E171 and Q were assessed by high-resolution respirometry (Oxygraph-2K; Oroboros Instruments, Innsbruck, Austria). Measurements were performed at 37 °C in air-saturated Krebs-Ringer medium containing 125 mM NaCl, 5 mM KCl, 25 mM HEPES, 1.4 mM CaCl_2_, 1 mM KH_2_PO_4_, 1 mM MgCl_2_ (pH 7.4), supplemented with 5 mM glucose. After recording routine (basal) respiration of H9c2 cells (0.3–1 mg protein/mL), oligomycin (final concentration 5 μM) was added to determine the oligomycin-insensitive respiration (LEAK). Once a steady state was reached, CCCP (final concentration 1 μM) was added to obtain maximal electron transport system (ETS) capacity. Finally, potassium cyanide (KCN, final concentration 10 mM) was added to completely inhibit mitochondrial oxygen consumption, allowing correction for residual oxygen consumption (ROX). All respiratory states were corrected by subtracting ROX. Oxidative phosphorylation-associated respiration (OxPhos) was calculated as routine respiration minus Leak.

### 2.7. TEM Analysis

After exposure to E171, H9c2 cells were fixed with 2.5% glutaraldehyde–paraformaldehyde in PBS for 45 min, followed by post-fixation in 1% osmium tetroxide for 1 h. Cells were dehydrated through a graded ethanol series and embedded in Epon 812 epoxy resin. Ultrathin sections (60 nm) were obtained using a diamond knife on an Ultracut-R ultramicrotome, mounted on copper grids, and contrasted with uranyl acetate and lead nitrate. Sections were examined using a JEOL 10/10 transmission electron microscope operated at 60 kV, and images were acquired with an Advanced Microscopy Techniques (AMT) Camera System (Woburn, Massachusetts, USA).

Mitochondrial morphology was evaluated according to predefined ultrastructural criteria. Mitochondria were classified as: (i) preserved (intact double membrane and well-defined cristae), (ii) without cristae (loss of internal cristae structure), (iii) ruptured (disruption of the outer mitochondrial membrane), or (iv) electron-dense (increased matrix density). Quantification was performed from independent cells in the experimental condition. A total of 20 mitochondria from 13 control cells and 17 mitochondria from 13 E171-treated cells were analyzed. Mitochondrial area was quantified using ImageJ software (Version ImageJ 2.16.0, National Institutes of Health (NIH), Bethesda, MD, USA). Individual mitochondria were manually delineated, and the cross-sectional area (µm^2^) was calculated for each structure.

### 2.8. Statistical Analysis

All experiments were performed in triplicate, and the data were analyzed as the mean ± standard deviation (SD). One-way analysis of variance, Tukey’s test, and Student’s *t*-test were used to determine the statistical significance of the experimental condition relative to the control. For mitochondrial ultrastructure analysis, statistical tests were performed using Fisher’s exact test for categorical variables and the Mann–Whitney test.

## 3. Results

### 3.1. E171 Increases Intracellular Labile Iron Levels in H9c2 Cardiomyoblasts

**Figure 1:**
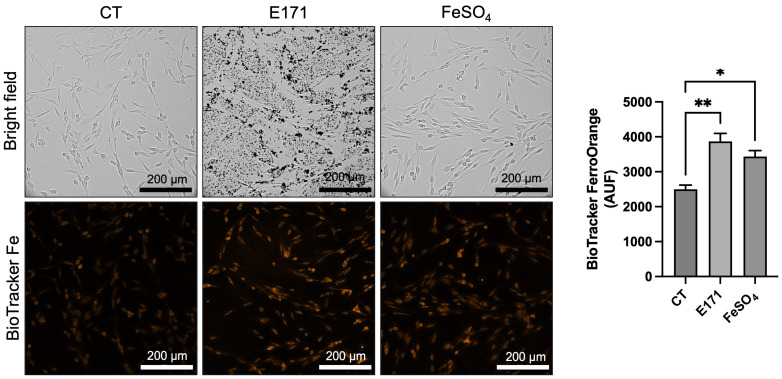
E171 increases intracellular ferrous iron levels in H9c2 cardiomyoblasts. Representative bright-field images and fluorescence micrographs showing intracellular iron levels detected using the fluorescent probe BioTracker^TM^ FerroOrange in H9c2 cells. Orange fluorescence corresponds to intracellular labile Fe^2+^ levels. Cells were exposed to E171 or treated with ferrous sulfate (FeSO_4_) as a positive control for Fe^2+^ accumulation. Quantification of fluorescence intensity (arbitrary fluorescence units, AUF) confirms a significant increase in intracellular labile Fe^2+^ levels following E171 exposure, with FeSO_4_ producing a comparable elevation consistent with its role as a positive control. Data are presented as mean ± SD (*n* = 3 independent experiments). Statistical significance was determined using one-way ANOVA followed by appropriate Tukey analysis (* *p* < 0.05, ** *p* < 0.005). CT = non-exposed cells.

To determine whether exposure to E171 alters intracellular labile iron levels, these levels were assessed using the fluorescent probe FerroOrange in H9c2 cardiomyoblasts. A 100 μM ferrous sulfate (FeSO_4_) for 24 h was used as a positive control for intracellular Fe^2+^ generation to validate the assay’s sensitivity. Cells exposed to E171 exhibited a significant increase in intracellular labile iron compared with control (CT) cells, as evidenced by enhanced fluorescence. As expected, treatment with FeSO_4_ resulted in a robust increase in fluorescence intensity, confirming the probe’s ability to detect intracellular ferrous iron (Figure 1). These findings indicate that E171 promotes the accumulation of labile Fe^2+^ in H9c2 cardiomyoblasts, suggesting disruption of its homeostasis.

### 3.2. E171 Promotes Oxidative Stress in H9c2 Cardiomyoblasts

**Figure 2:**
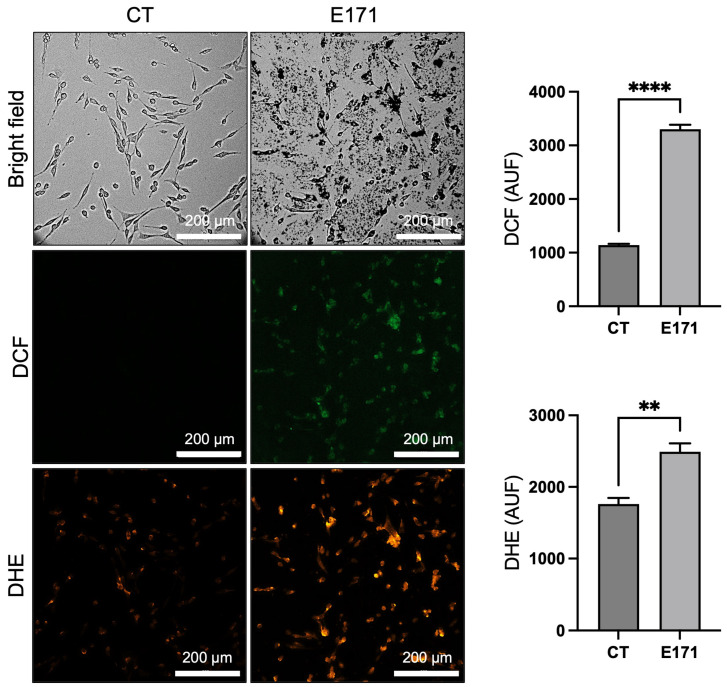
E171 increases ROS production in H9c2 cardiomyoblasts. Representative bright-field and fluorescence images showing intracellular ROS levels in H9c2 cells following exposure to E171. ROS generation was assessed using fluorescent probes for general oxidative species (DCF) and for detecting superoxide anion (DHE). Cells were stained with H_2_DCFDA, where green fluorescence reflects intracellular ROS generation, and with dihydroethidium (DHE), where red fluorescence indicates intracellular superoxide (O_2_•^−^) production. Fluorescence microscopy revealed a marked increase in ROS levels in cells treated with E171 compared with control cells. Quantification of fluorescence intensity (arbitrary fluorescence units, AFU) confirmed a significant increase in both DCF and DHE signals following E171 exposure. Data are presented as mean ± SD from 3 independent experiments. Statistical significance was determined using Student’s *t*-test (** *p* < 0.001; **** *p* < 0.0001). CT = non-exposed cells.

Given the augmentation of intracellular iron levels, we linked this scenario to increased oxidative stress in H9c2 cardiomyoblasts exposed to E171. Intracellular ROS production was assessed using the fluorescent probes DHE and H_2_DCFDA. Fluorescence microscopy revealed a marked increase in DCF signal in E171-treated cells compared with CT cells. Quantitative analysis confirmed this response, a significant increase in DCF fluorescence intensity following E171 exposure. Similarly, O_2_•^−^, as assessed by DHE staining, was significantly increased in cells exposed to E171 relative to control cells (Figure 2). These results demonstrate that E171 induces a pronounced oxidative stress response in H9c2 cardiomyoblasts.

### 3.3. E171 Alters the Cellular Redox State

**Figure 3:**
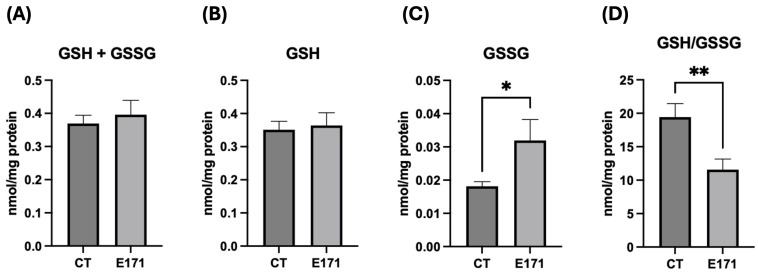
E171 disrupts glutathione redox homeostasis in H9c2 cardiomyoblasts. Intracellular glutathione levels were quantified in H9c2 cells following exposure to E171. (**A**) Total glutathione levels (GSH + GSSG), (**B**) reduced glutathione (GSH), (**C**) oxidized glutathione (GSSG), and (**D**) the GSH/GSSG ratio were determined and normalized to protein content. While GSH + GSSG and GSH levels were not significantly altered compared with control conditions, exposure to E171 significantly increased GSSG. Consequently, the GSH/GSSG ratio was markedly reduced, indicating disruption of the cellular redox balance. Data are presented as mean ± SD from 3 independent experiments. Statistical significance was determined using Student’s *t*-test (* *p* < 0.05, ** *p* < 0.005).

To further determine whether iron disruption and ROS production induced by E171 affect the redox state, we evaluated the GSH + GSSG, GSH, GSSG, and the GSH/GSSG ratio in H9c2 cardiomyoblasts. As shown in Figure 3, GSH + GSSG were not significantly altered following E171 exposure. Similarly, GSH levels remained comparable to those observed in CT cells. In contrast, GSSG levels were significantly increased in cells treated with E171. Consistent with this observation, the GSH/GSSG ratio was markedly reduced in this last group compared with CT conditions. These results indicate that exposure to E171 promotes oxidation of the intracellular GSH pool, disrupting the cellular redox balance in H9c2 cardiomyoblasts.

### 3.4. E171 Induces Mitochondrial Ultrastructural Alterations in H9c2 Cardiomyoblasts

**Figure 4:**
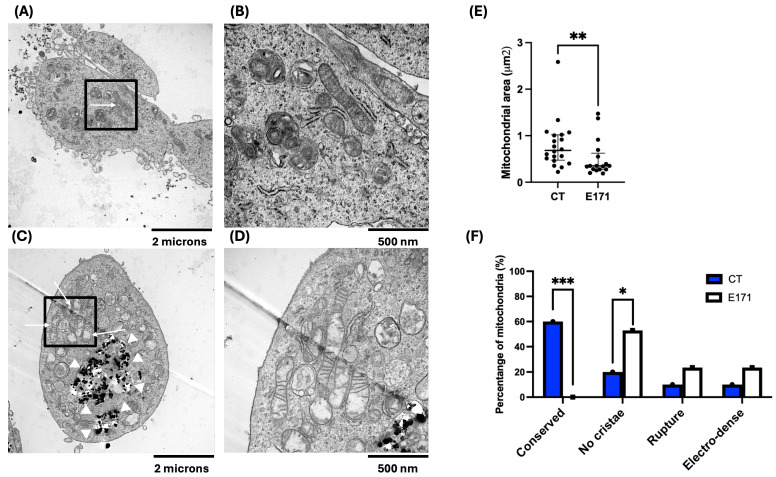
E171 induces mitochondrial ultrastructural alterations in H9c2 cardiomyoblasts. Transmission electron microscopy (TEM) micrographs showing representative mitochondrial morphology in control (CT) and E171-treated cells. (**A**) Control cells exhibiting preserved mitochondrial ultrastructure. (**B**) Higher magnification of control mitochondria displaying intact outer membranes and well-defined cristae. (**C**) E171-treated cells showed accumulation of electron-dense aggregates and altered mitochondrial morphology. (**D**) Higher magnification of E171-treated cells demonstrates mitochondrial abnormalities, including loss of cristae, membrane rupture, and increased electron density. Boxes indicate the areas enlarged in the high-magnification TEM images shown in panels B and D. (**E**) Quantification of mitochondrial area measured from TEM micrographs. The mitochondrial area was measured using ImageJ software. (**F**) Percentage distribution of mitochondria classified as conserved, without cristae (No-cristae), membrane rupture, or electron-dense. Percentages were calculated relative to the total number of mitochondria analyzed per group. A total of 20 mitochondria from 13 independent cells (CT) and 17 mitochondria from 13 independent cells (E171) were evaluated. In the TEM sections analyzed, only one E171-treated cell contained three mitochondria suitable for morphometric evaluation; all other cells contributed one or two mitochondria. Statistical analysis was performed using Fisher’s exact test for categorical variables and the Mann–Whitney test. * *p* < 0.05; ** *p* < 0.005, *** *p* < 0.0005 versus CT. Scale bars: (**A**,**C**) 2 µm; (**B**,**D**) 500 nm. Arrows indicate mitochondria. Arrowheads indicate E171 nanoparticles (NPs).

Given the observed iron accumulation, increased ROS, and disruption of GSH/GSSG status, we next examined whether exposure to E171 was associated with alterations in the mitochondrial ultrastructure. TEM analysis revealed clear morphological differences between control and E171-treated cells (Figure 4). In control cells, mitochondria displayed normal morphology characterized by well-defined membranes and preserved cristae organization. In contrast, cells exposed to E171 exhibited evident mitochondrial abnormalities, including loss of cristae organization, membrane rupture, increased electron density, and rounded mitochondria with altered internal structure. To objectively quantify these alterations, morphometric analysis was performed using ImageJ software. E171 exposure significantly altered mitochondrial area compared with control cells (Figure 4E; *p* < 0.005). Furthermore, quantitative classification of mitochondrial morphology revealed a significant reduction in the percentage of conserved mitochondria in E171-treated cells, accompanied by a significant increase in mitochondria lacking cristae (Figure 4F; *p* < 0.0005, *p* < 0.05). No statistically significant differences were observed in the proportion of mitochondria exhibiting membrane rupture or electron-dense morphology between groups. These quantitative findings confirm that E171 exposure induces measurable mitochondrial structural remodeling in H9c2 cardiomyoblasts. Thus, these observations indicate that exposure to E171 is associated with significant mitochondrial structural damage in H9c2 cardiomyoblasts.

### 3.5. E171 Impairs Mitochondrial Respiratory Function in H9c2 Cardiomyoblasts

**Figure 5:**
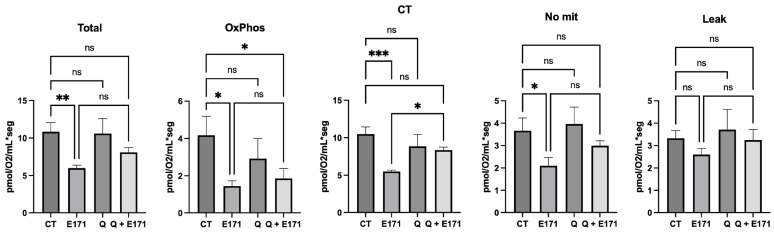
E171 impairs mitochondrial respiration in H9c2 cardiomyoblasts. Mitochondrial respiratory function was evaluated in H9c2 cells by oxygen consumption rate (OCR) parameters under control conditions (CT), in the presence of Quercetin (Q), after exposure to E171, and in cells treated with both quercetin and E171 (Q + E171). Respiratory parameters analyzed included total respiration, oxidative phosphorylation (OxPhos), electron transport system capacity (chain transporter (CT)), no mitochondrial (No mit), and Leak. Exposure to E171 significantly reduced several mitochondrial respiratory parameters compared with control conditions, indicating impaired mitochondrial bioenergetics. Q treatment alone did not significantly affect mitochondrial respiration or prevent mitochondrial dysfunction induced by nanoparticle exposure. Data are presented as mean ± SD (n = 3 independent experiments). Statistical significance was determined using one-way ANOVA followed by appropriate Tukey analysis (* *p* < 0.05, ** *p* < 0.005, *** *p* < 0.0005). ns = no significant.

To determine whether the mitochondrial ultrastructural alterations were associated with functional impairment, mitochondrial respiration was evaluated in H9c2 cardiomyoblasts. Oxygen consumption rate (OCR) parameters were analyzed in all our experimental conditions. Under control (CT) conditions, exposure to E171, in the presence of 10 μM Q (which has been shown to significantly reduce ROS and iron levels, preserving mitochondrial function [11,13,14]), was followed by cells treated with both quercetin and E171 (Q + E171). Exposure to E171 significantly reduced total mitochondrial respiration compared with CT cells (Figure 5). A similar reduction was observed in oxidative phosphorylation–linked respiration (OxPhos) and electron transport chain capacity (CT), indicating impairment of mitochondrial respiratory activity. Additionally, E171 exposure significantly decreased non-mitochondrial respiration (No mit) parameters, whereas leak respiration was not significantly affected. Treatment with Q alone did not significantly alter mitochondrial respiration relative to control conditions. Moreover, Q did not restore the respiratory impairment induced by E171 at the evaluated concentration and exposure conditions, as respiratory parameters in the Q + E171 group remained significantly reduced compared with control cells. These findings indicate that exposure to titanium dioxide compromises mitochondrial respiratory function in H9c2 cardiomyoblasts and that Q treatment does not rescue this mitochondrial dysfunction under the experimental conditions tested.

## 4. Discussion

E171 is one of the most widely used food additives worldwide, and it can reach high concentrations in the human body. In the USA, the population ingests around 0.15–3.9 mg/kg body weight/day. A 2024 study reported that the Chinese population ingests, on average, 34.84 mg/kg body weight/day of E171, with children under 10 years of age consuming the highest amount, reaching up to 90.27 mg/kg body weight/day. Meanwhile, the population aged 10 to 17 years ingests around 52.24 mg/kg body weight/day, and adults aged 18 to 64 years consume an average of 26.36 mg/kg body weight/day [15]. In Europe, it is estimated that children ingest between 3.89 and 16.6 mg/kg/day [16]. The concentration of E171 used in H9c2 cells is not comparable to the concentrations consumed in humans; however, E171 can accumulate in cardiac cells with continuous exposure, causing irreversible damage [17]. Despite several toxic effects reported for this additive, in 2021, the European Food Safety Authority (EFSA) published a new safety assessment and concluded that “E171 can no longer be considered safe when used as a food additive” due to its genotoxic effects [18]. In many countries, there are no important regulations for its use. Since they contain a nanometric fraction of TiO_2_, human exposure to these nanoparticles through oral ingestion can be very dangerous, raising questions about their health effects.

The present study demonstrates that exposure to E171 significantly increased intracellular labile Fe^2+^ levels, as detected by FerroOrange fluorescence, indicating an elevation in the redox-active iron pool. The latter promotes oxidative stress and could impair mitochondrial ultrastructure and function in H9c2 cardiomyoblasts, thereby contributing to a redox imbalance that ultimately compromises mitochondrial bioenergetics.

The observed increase in labile Fe^2+^ suggests that E171 exposure alters intracellular iron dynamics, thereby expanding the redox-active iron pool. However, we did not assess iron-regulatory proteins such as ferritin, transferrin, or transferrin receptor 1 (TfR1), which would be necessary to fully characterize alterations in iron homeostasis. Therefore, our findings should be interpreted as evidence of increased labile iron rather than a comprehensive disruption of iron regulatory pathways.

In addition, although increased Fe^2+^ and ROS levels were observed concomitantly with mitochondrial ultrastructural alterations, the present study does not establish a direct causal relationship between iron accumulation and mitochondrial dysfunction. Interventional approaches, such as iron chelation, will be required in future studies to determine whether iron dysregulation mechanistically contributes to the mitochondrial damage induced by E171.

Previous studies conducted by our group have shown that this food additive is taken up by H9c2 cells, leading to increased ROS production, alterations in mitochondrial membrane potential associated with oxidative stress, and mitochondrial alterations [19,20]. Therefore, we explored one of the main mechanisms possibly associated with these effects: the intracellular iron levels. One of the earliest alterations observed in this study in H9c2 cells exposed to E171 was the accumulation of intracellular ferrous iron (Fe^2+^). Iron plays a critical role in cellular metabolism; however, dysregulation of intracellular iron levels can promote the generation of ROS through Fenton-type reactions. Previous studies have reported that metal-based nanoparticles can interfere with cellular iron metabolism and contribute to oxidative stress [21]. In this context, the increase in intracellular iron observed here may represent an important upstream event contributing to the oxidative imbalance induced by E171.

Although we observed a significant increase in intracellular Fe^2+^ levels following chronic E171 exposure, the precise upstream mechanism responsible for disrupting iron homeostasis remains to be determined. It is unclear whether E171 promotes iron release from intracellular storage proteins such as ferritin, enhances iron uptake through membrane transporters, or impairs iron export. Future studies that address the expression and regulation of iron-handling proteins will be necessary to define the molecular basis of labile iron accumulation in this model.

Consistent with this interpretation, the increased ROS production observed under experimental conditions was consistent with previous reports showing that E171 can induce oxidative stress in different cell types, such as Caco-2 intestinal epithelial cells or HT29-MTX mucus-secreting cells [22]. Oxidative stress is widely recognized as a central mechanism underlying the toxicity of titanium dioxide particles and has been associated with mitochondrial damage and altered cellular metabolism.

Further supporting the presence of oxidative stress, our results show a disruption of the glutathione redox balance. While GSH + GSSG and GSH levels remained relatively stable, GSSG levels increased significantly, leading to a marked reduction in the GSH/GSSG ratio. This shift indicates oxidation of the intracellular GSH pool and reflects a compromised cellular antioxidant defense system. The GSH/GSSG ratio is widely considered a sensitive indicator of cellular redox state, and its reduction suggests that E171 overwhelms the antioxidant buffering capacity of cardiomyoblasts.

Given mitochondria’s central role in cellular redox regulation and energy metabolism, we next examined mitochondrial ultrastructure. TEM revealed clear mitochondrial alterations in E171-treated cells, including swelling and disruption of cristae organization. Such structural abnormalities are frequently associated with mitochondrial dysfunction and impaired oxidative metabolism. Mitochondria are not only central organelles in metabolism and energy conversion but are also platforms for cellular signaling cascades. Alterations in mitochondrial shape and ultrastructure have been observed during cell death and human diseases, and these dynamic changes in mitochondrial morphology can, in turn, modulate mitochondrial function, thereby inducing dysfunction [23]. Cristae organization is dynamic, allowing it to respond to metabolic and bioenergetic demands by controlling the formation and stability of respiratory supercomplexes (RSCs) and ATP synthase oligomers. Significant changes in cristae were observed in H9c2 cells exposed to E171, compared with control cells. Cristae rearrangements, such as tightening, cause the release of nearly 80% of cytochrome c (Cyt-c) confined within the intracristal space, thereby inducing apoptosis [24]. Mitochondrial alterations decreased, intracellular Cyt-c levels dropped, and apoptosis was observed in hearts from rats chronically exposed to E171 [20,25], results that coincide with those found in this study. The structural disruption of mitochondrial cristae observed in E171-treated cells may compromise respiratory efficiency and could potentially predispose cells to apoptotic signaling. Although apoptotic pathways were not directly assessed in the present in vitro study, previous in vivo work from our group demonstrated that oral exposure to food-grade titanium dioxide nanoparticles induces cardiac damage and apoptosis in rat models [25]. Therefore, the mitochondrial structural alterations and bioenergetic impairment observed here may represent early upstream events that precede activation of apoptotic signaling under prolonged or systemic exposure conditions.

Although in vitro concentrations cannot be directly translated to human dietary intake levels, repeated exposure to E171 through food consumption raises concerns regarding potential cumulative effects. Future in vivo studies examining long-term particle biodistribution, cardiac accumulation, and bioenergetic consequences will be necessary to determine whether chronic exposure leads to progressive cardiac dysfunction beyond the acute effects observed in this cellular model. Consistent with these structural alterations found in H9c2 treated with E171, mitochondrial respiration analysis revealed significant reductions in several respiratory parameters following E171 exposure. Total respiration, oxidative phosphorylation–linked respiration, and electron transport system capacity were markedly decreased, indicating compromised mitochondrial bioenergetics. These findings suggest that E171 impairs mitochondrial function at multiple levels, ultimately reducing cells’ capacity to sustain efficient oxidative phosphorylation.

Interestingly, E171 exposure also reduced non-mitochondrial oxygen consumption. Although this parameter is often considered a residual component in mitochondrial stress assays, changes in non-mitochondrial respiration may reflect alterations in cytosolic oxidase activity, redox enzymes, or other oxygen-consuming processes beyond oxidative phosphorylation. This observation suggests that the metabolic impact of E171 may extend beyond mitochondrial bioenergetics, potentially affecting broader cellular redox and metabolic pathways.

However, the primary aim of the present study was to characterize alterations in labile iron levels, oxidative stress, and mitochondrial bioenergetic function. A comprehensive evaluation of global metabolic remodeling would require targeted metabolomics or multi-omics approaches, which were beyond the scope of this study. Future investigations addressing these aspects may provide deeper insight into the systemic metabolic consequences of E171 exposure in cardiomyocytes. 

Q was included in this study to evaluate whether antioxidant supplementation could mitigate nanoparticle-induced mitochondrial dysfunction. The concentration of 10 μM was selected based on previously published in vitro studies demonstrating that low-micromolar concentrations of quercetin exert antioxidant and cytoprotective effects without inducing cytotoxicity. In H9c2 cardiomyoblasts, 10 μM Q has been shown to significantly reduce intracellular ROS levels, preserve mitochondrial function, and attenuate oxidative stress-induced cellular damage [11]. In addition to its radical-scavenging activity, Q possesses metal-binding properties due to its catechol and 3-hydroxy-4-keto structural motifs, allowing it to chelate redox-active iron and potentially modulate the intracellular labile iron pool [13]. Concentrations within the 5–20 μM range of Q have been reported to modulate redox homeostasis and reduce iron-driven oxidative reactions in cellular systems [26]. Therefore, 10 μM Q was selected as a biologically relevant concentration to evaluate both the antioxidant and iron-modulating potential of Q in the context of titanium dioxide nanoparticle-induced oxidative stress. Paradoxically, under the experimental conditions tested (10 μM, single time point), Q did not significantly improve the evaluated mitochondrial parameters in cells exposed to E171. Although Q is widely recognized for its antioxidant properties and mitochondrial-protective effects in cardiomyoblasts, our data indicate that, under the conditions tested, it was insufficient to counteract mitochondrial dysfunction. A 10 μM Q treatment did not restore mitochondrial bioenergetics following E171 exposure. The selected concentration was based on previous in vitro studies in H9c2 cells demonstrating that low-micromolar Q (5–20 μM) reduces ROS levels, preserves mitochondrial membrane potential, and attenuates oxidative stress without inducing cytotoxicity. Moreover, since Q possesses iron-chelating properties due to its catechol and 3-hydroxy-4-keto structural motifs, supports its potential to modulate the intracellular labile iron pool. This observation suggests that E171-induced mitochondrial damage may involve mechanisms beyond simple oxidative stress, potentially including direct interactions with mitochondrial components or persistent disturbances in cellular redox balance. Since Q has been reported to exert mitochondrial-protective effects in other models, only a single concentration (10 μM) and a single time point were evaluated in this study. Therefore, the absence of a significant protective effect should be interpreted within the context of the specific experimental conditions employed. Additional dose–response and time-course studies, as well as assessment of intracellular quercetin accumulation, are warranted to fully elucidate its potential protective capacity against E171-induced mitochondrial alterations. Thus, the absence of a significant protective effect should be interpreted within the context of the specific experimental conditions employed in this study.

The failure of Q to restore mitochondrial respiration, despite its recognized antioxidant and iron-chelating properties, suggests that E171-induced mitochondrial dysfunction may not be exclusively mediated by reversible oxidative stress. It is plausible that E171 particles exert direct physicochemical interactions with mitochondrial membranes or respiratory chain complexes, potentially leading to structural alterations that persist beyond redox normalization. In this context, the disruption of cristae architecture observed by transmission electron microscopy may reflect deeper impairment of mitochondrial organization. Although the present study did not assess mitochondrial DNA integrity or individual respiratory complex activities, such analyses would be valuable for determining whether irreversible mitochondrial damage contributes to the sustained bioenergetic deficit observed after E171 exposure.

E171 is a heterogeneous material composed of both nano- and micro-sized TiO_2_ particles. In the present study, we evaluated the commercial preparation as a whole to mimic realistic exposure conditions. However, it remains unclear whether the observed iron dysregulation and mitochondrial dysfunction are predominantly driven by the nanoscale fraction, which may exhibit enhanced cellular penetration, or whether synergistic interactions between nano- and microparticles contribute to the observed toxicity. Future investigations that separate these fractions and characterize their intracellular distribution and bioenergetic impact will be necessary to delineate their relative contributions.

These findings highlight the complexity of nanoparticle-induced mitochondrial injury and suggest that antioxidant intervention alone may not be sufficient to fully restore mitochondrial bioenergetics once structural damage is established.

**Figure 6:**
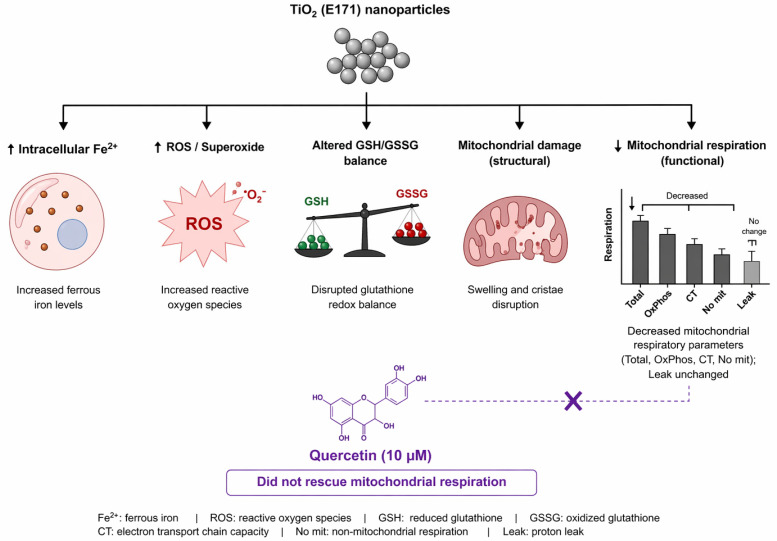
Integrative schematic representation of the effects of E171 on H9c2 cardiomyoblasts. Exposure to E171 increased intracellular ferrous iron (Fe^2+^) levels and reactive oxygen species (ROS), disrupted the glutathione redox balance (increased GSSG and decreased GSH/GSSG ratio), and induced mitochondrial structural alterations characterized by swelling and cristae disorganization. Functionally, mitochondrial respiration was significantly reduced, as evidenced by decreased total respiration, oxidative phosphorylation (OxPhos), electron transport chain capacity (CT), and non-mitochondrial respiration (No mit), while proton leak remained unchanged. Treatment with (Q, 10 μM) did not significantly ameliorate mitochondrial ultrastructural alterations or mitochondrial respiratory parameters under the experimental conditions tested.

Taken together, the present findings suggest that E171 promotes a cascade of events characterized by labile iron accumulation, oxidative stress, disruption of glutathione homeostasis, and mitochondrial structural and functional impairment (Figure 6). These alterations may contribute to cardiomyocyte vulnerability under conditions of nanoparticle exposure.

## 5. Conclusions

In conclusion, our study provides evidence that E171 increases intracellular labile Fe^2+^ levels, induces oxidative stress, and compromises mitochondrial integrity and bioenergetic function in H9c2 cardiomyoblasts, highlighting the potential impact of exposure to this food additive on cardiac cellular metabolism. Treatment with Q 10 μM did not restore mitochondrial bioenergetics after exposure to E171. Further studies will be required to clarify the precise molecular mechanisms underlying these alterations and to determine their relevance in vivo.

## Data Availability

The original contributions presented in this study are included in the article/Supplementary Material. Further inquiries can be directed to the corresponding author.

## Supplementary Materials

The following supporting information can be downloaded at https://www.mdpi.com/article/10.3390/jox16030108/s1, Figure S1:Representative bright-field images and fluorescence micrographs showing intracellular iron levels detected using the fluorescent probe BioTrackerTM FerroOrange in H9c2 cells. Figure S2: Representative bright-field and fluorescence images showing intracellular ROS levels in H9c2 cells. Figure S3: Transmission electron microscopy (TEM) micrographs showing representative mitochondrial morphology in control (CT) and E171-treated cells.

## Abbreviations

The following abbreviations are used in this manuscript:

E171	titanium dioxide
TEM	transmission electron microscopy
ROS	reactive oxygen species
KCl	Potassium chloride
HEPES	ethanesulfonic acid
CaCl_2_	calcium chloride
MgCl_2_	magnesium chloride
DTNB	5,5-dithio-bis (2 nitrobenzoic) acid
GSH	glutathione reduced
GSSG	glutathione oxidized
GR	glutathione reductase
DHE	dihydroethidium
H_2_DCFDA	2,7-dichlorofluorescin diacetate
K_2_HPO_4_	potassium phosphate
DPI	diphenylene iodonium
CCCP	carbonyl cyanide chlorophenyl hydrazone
NADH	nicotinamide adenine dinucleotide
DMSO	dimethyl sulfoxide
Na_2_HPO_4_	sodium phosphate dibasic
NaH_2_PO_4_	sodium phosphate monobasic
2-VP	2-vinylpyridine
FeSO_4_	ferrous sulfate
Q	quercetin
DMEM	Dulbecco’s Modified Eagle’s Medium
FBS	Fetal bovine serum
ATCC	American Type Culture Collection
CO_2_	carbon dioxide
Fe^2+^	labile iron (II) ions
Fe^3+^	iron (III) ions
PBS	phosphate-buffered solution
DCF	dichlorofluorescein
GSH + GSSG	Total glutathione
DTNB	5,5′-dithiobis(2-nitrobenzoic acid)
TNB	5-thio-2-nitrobenzoic acid
OCR	Oxygen consumption rates
ROX	residual oxygen consumption
OxPhos	Oxidative phosphorylation-associated respiration
SD	standard deviation
CT	control
No mit	non-mitochondrial respiration
EFSA	European Food Safety Authority
AMT	Advanced Microscopy Techniques
NIH	National Institutes of Health
Cyt-c	cytochrome c
RSCs	respiratory supercomplexes
