# Supplementary material for: Food Additive Titanium Dioxide (E171) Increases Intracellular Labile Fe2+ Levels and Induces Oxidative Stress and Mitochondrial Dysfunction in H9c2 Cardiomyoblasts
Source: J Xenobiot. 2026 Jun 9;16(3):108. doi: 10.3390/jox16030108 (PMC13301620; doi:10.3390/jox16030108)
Supplement: Supplementary file 1 [file jox-16-00108-s001.zip › jox-4306342-supplementary.pdf]

Journal of Xenobiotics

Supplementary Materials: Food Additive Titanium Dioxide (E171) Increases Intracellular Labile Fe2+ Levels and Induces Oxidative Stress and Mitochondrial Dysfunction in H9c2 Cardiomyoblasts

Alfredo Cruz-Gregorio, Alejandro Silva-Palacios, Javier A. Belmont-Díaz, María del Pilar Ramos-Godinez and Rebeca López-Marure

(A) CT (Bright field)

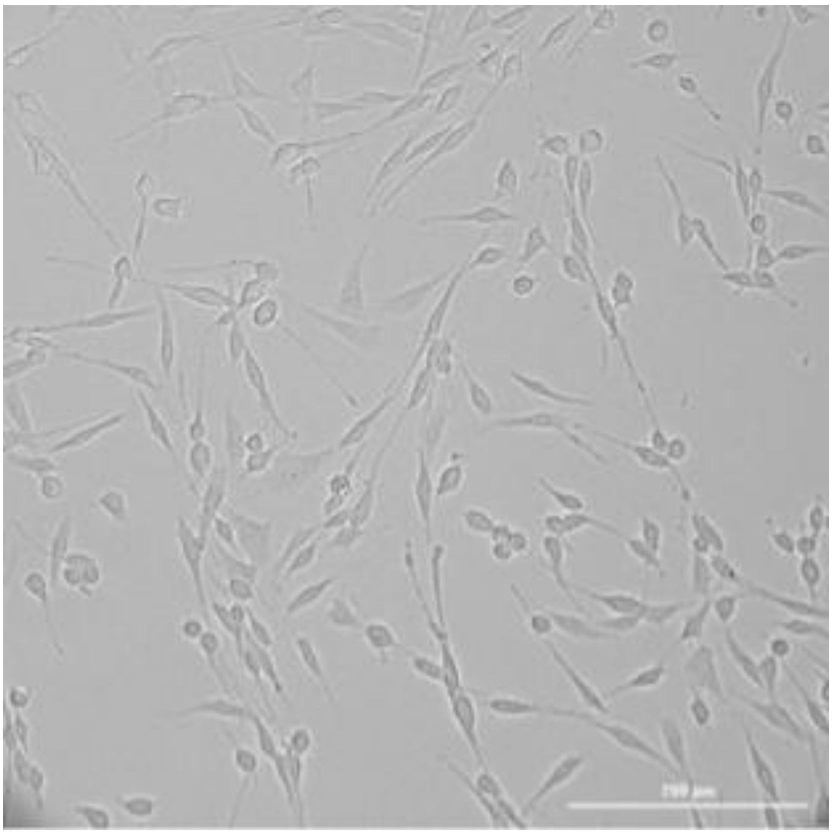

(B) CT (Biotracker Fe)

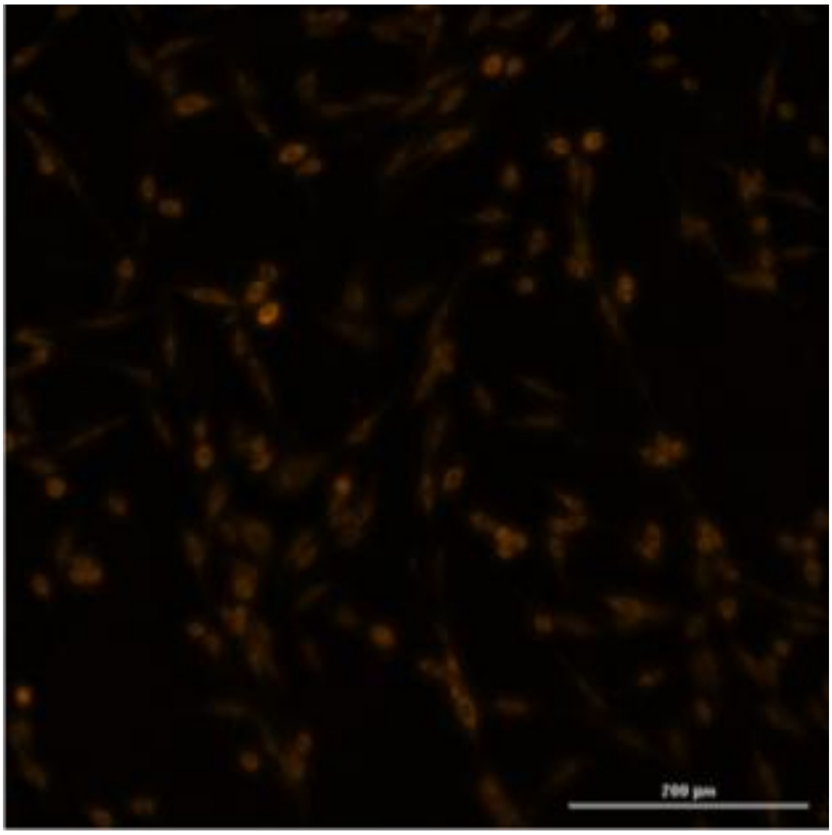

(C) E171 (Bright field)

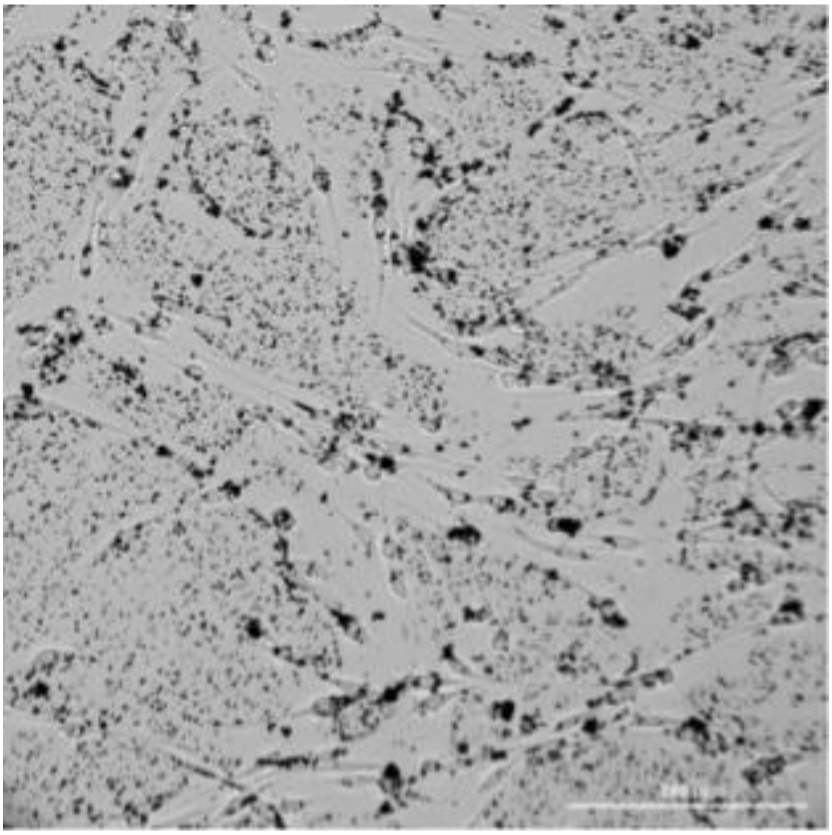

(D) E171 (Biotracker Fe)

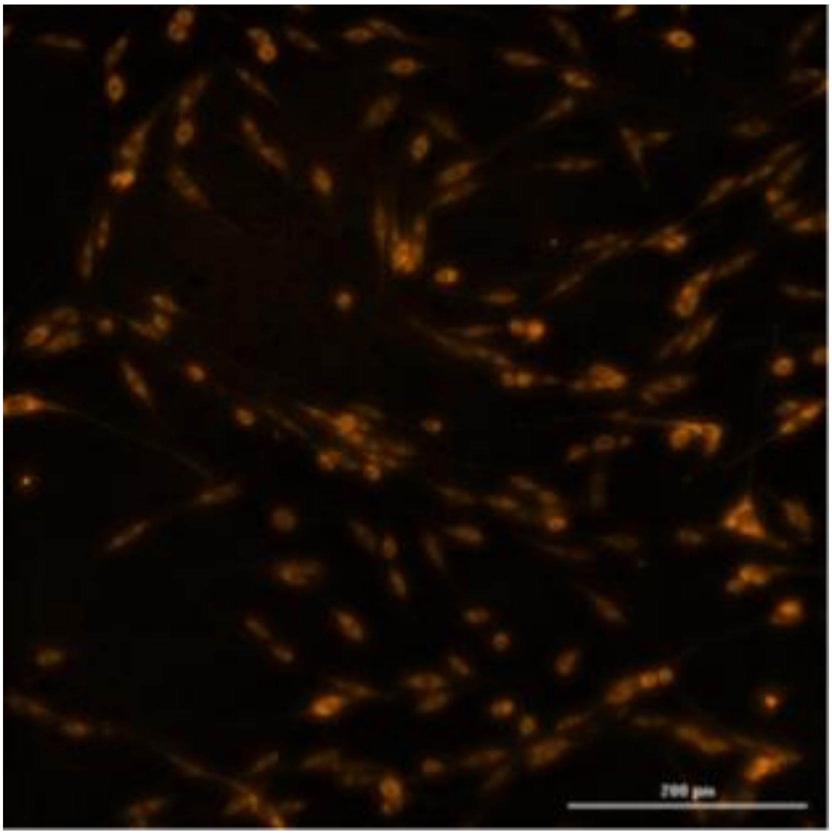

(E) $FeSO_4$ (Bright field)

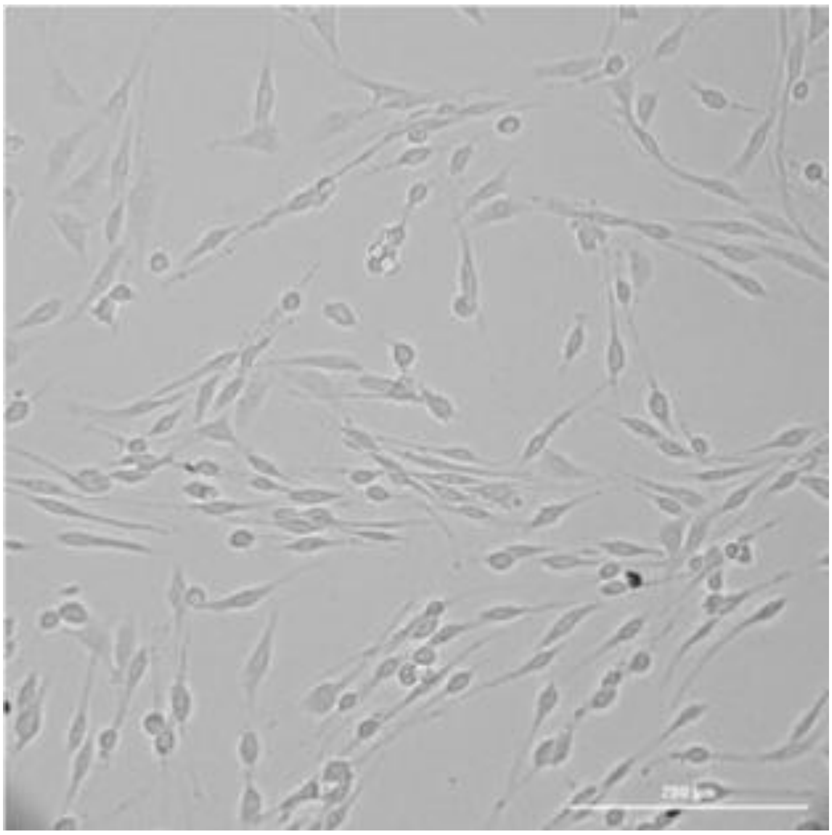

(F) $FeSO_4$ (Biotracker Fe)

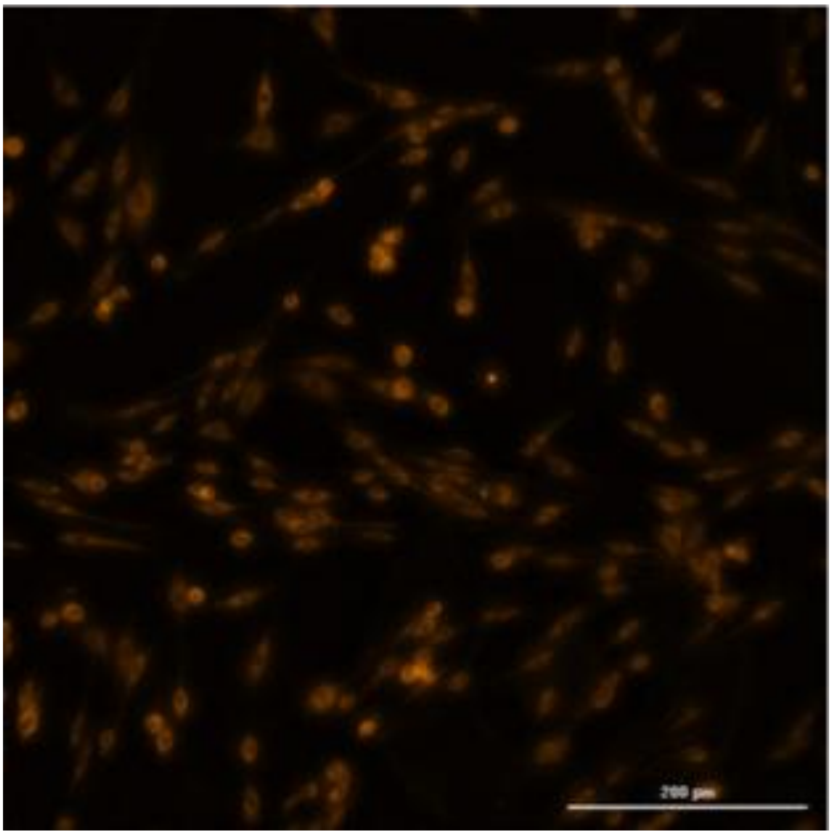

Figure S1: Representative bright-field images and fluorescence micrographs showing intracellular iron levels detected using the fluorescent probe BioTracker™ FerroOrange in H9c2 cells. (A, B) CT: Control cells, (C, D) E171: cells exposed to E171 or (E, F) treated with ferrous sulfate ($FeSO_4$) as a positive control for Fe^{2+} accumulation. Uncropped and unlabeled original images from Figure 1.

(A) CT (Bright field)

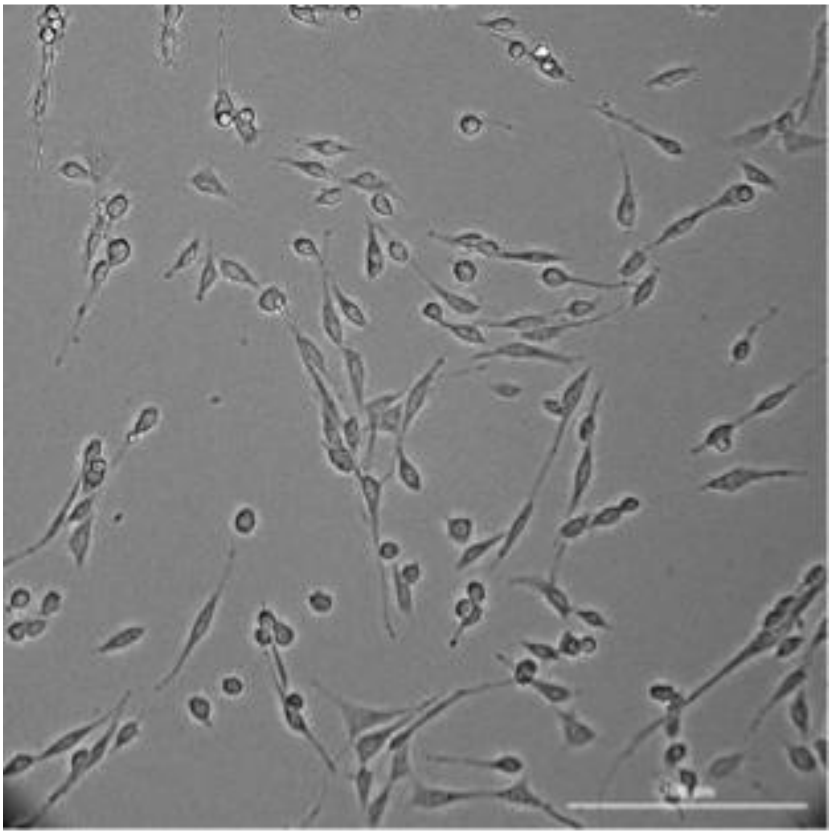

(B) CT (DCF)

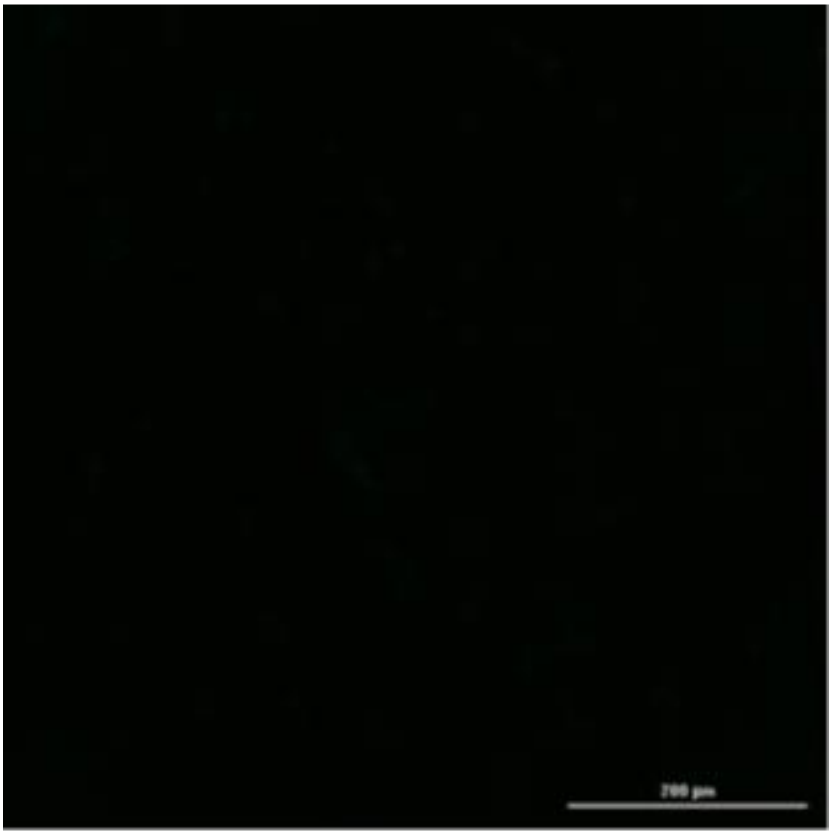

(C) CT (DHE)

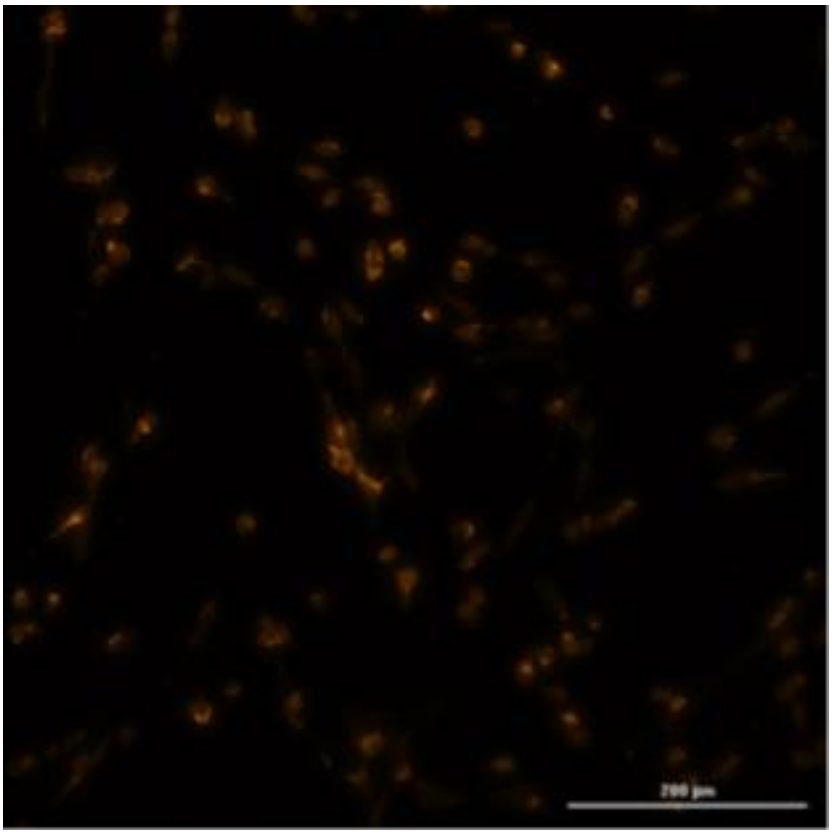

(D) E171 (Bright field)

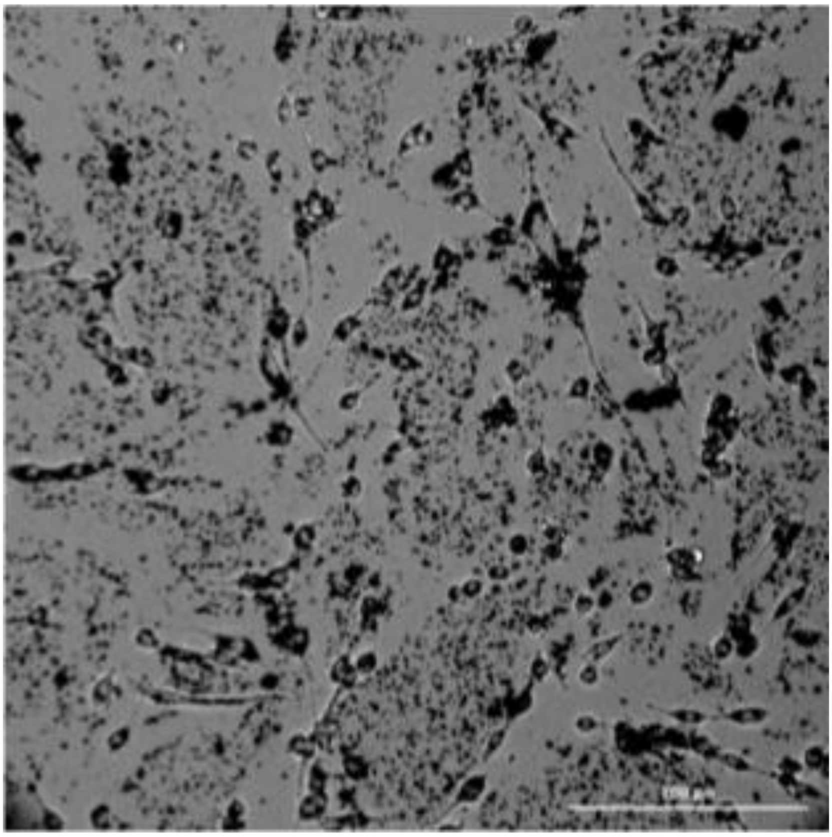

(E) E171 (DCF)

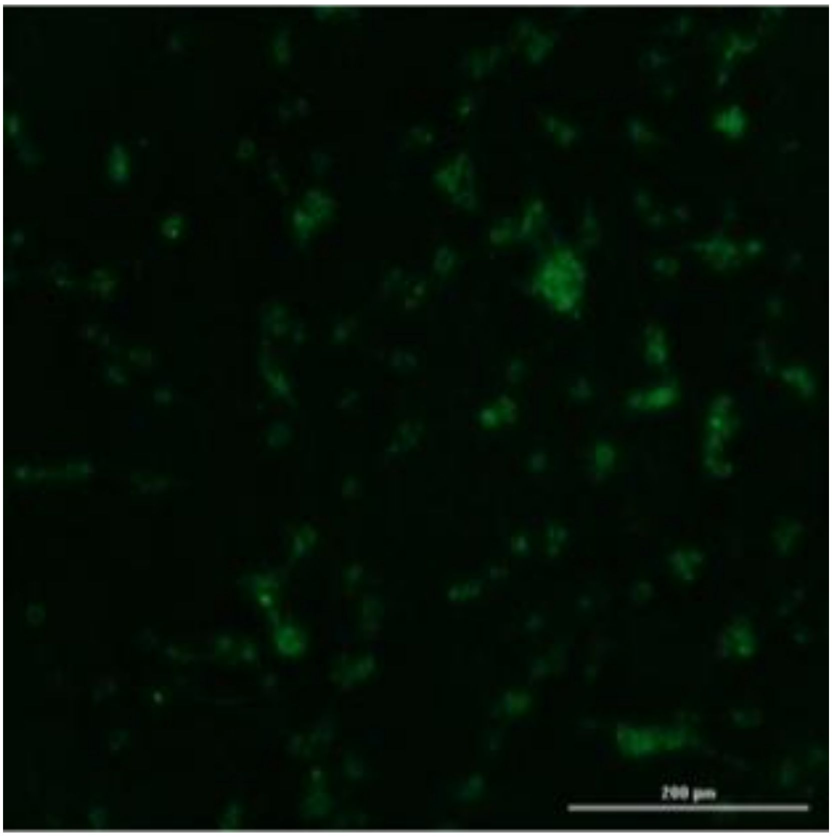

(F) E171 (DHE)

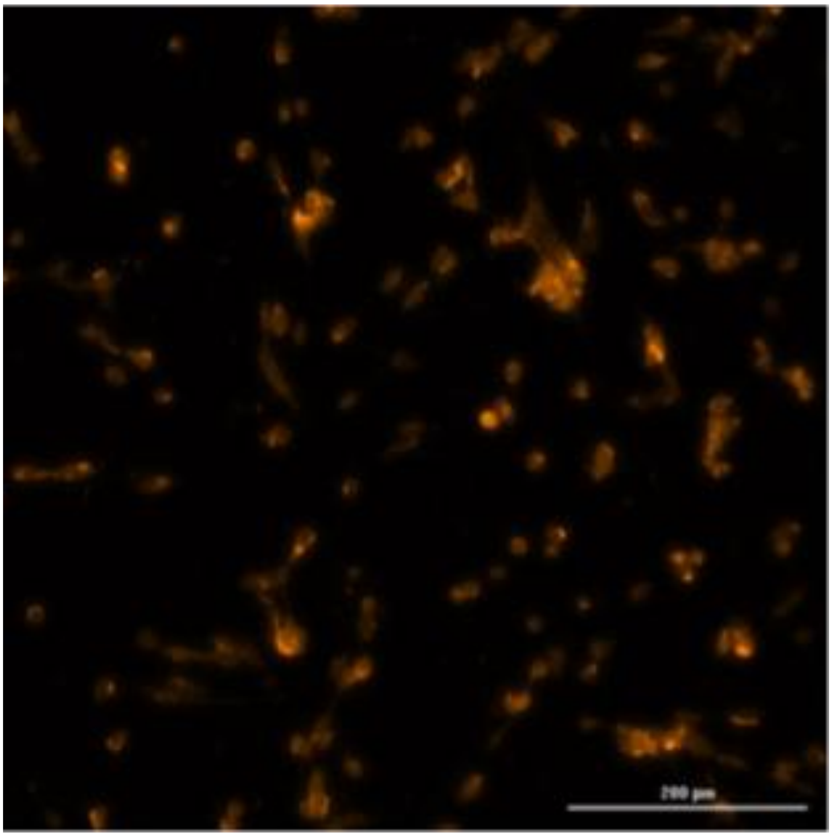

Figure S2: Representative bright-field and fluorescence images showing intracellular ROS levels in H9c2 cells. (A, B, C) CT, control cells and (D, E, F) cells exposed to E171. ROS generation was assessed using fluorescent probes for general reactive oxidative species (DCF) and for detecting superoxide anion (DHE). Uncropped and unlabeled original images from Figure 2.

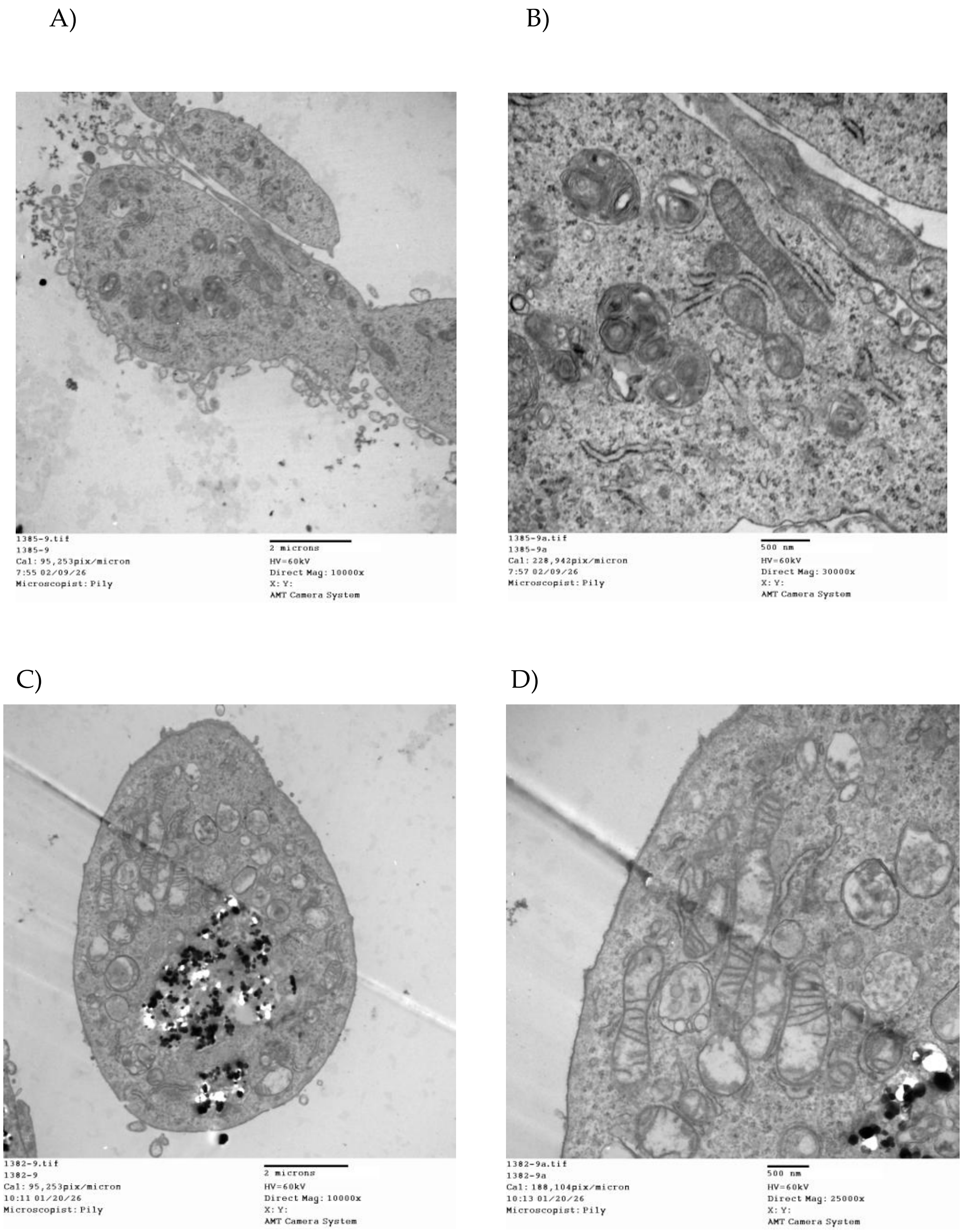

Figure S3: Transmission electron microscopy (TEM) micrographs showing representative mitochondrial morphology in control (CT) and E171-treated cells. (A) Control cells exhibiting preserved mitochondrial ultrastructure. (B) Higher magnification of control mitochondria displaying intact outer membranes and well-defined cristae. (C) E171-treated cells showed accumulation of electron-dense aggregates and altered mitochondrial morphology. (D) Higher magnification of E171-treated cells demonstrates mitochondrial abnormalities, including loss of cristae, membrane rupture, and in-creased electron density. Uncropped and unlabeled original images from Figure 3.
